# Supplementary material for: Complexome profiling of the Chlamydomonas psb28 mutant reveals TEF5 as an early PSII assembly factor
Source: Plant Cell. 2025 Mar 17;37(6):koaf055. doi: 10.1093/plcell/koaf055 (PMC12164590; doi:10.1093/plcell/koaf055)
Supplement: koaf055_Supplementary_Data [file koaf055_supplementary_data.zip › psb28_interactive.zip!/plots/intensity/qualityPlots/peptides/MGE1_Cre08.g370450.t1.2.html]

### Cre08.g370450.t1.2

Trivial name: MGE1  
SeqCoverage [median]: 42.80  
Mass: 28.42 kDa  
  
 

| Sequence | MissedCleavage | Mass [Da] | wtIntensity | muIntensity | Score |
| --- | --- | --- | --- | --- | --- |
| AASVVPSEALK | 0 | 1070.60 | 2909.98 | 9463.84 | 120.99 |
| AASVVPSEALKEDGGVPPEK | 1 | 1979.02 | 0.00 | 1274.40 | 79.31 |
| AASVVPSEALKEDGGVPPEKLR | 2 | 2248.21 | 680.97 | 1401.60 | 63.58 |
| ATESILHK | 0 | 897.49 | 984.02 | 810.26 | 129.55 |
| ENNTIAVVTK | 0 | 1087.59 | 0.00 | 1876.51 | 71.35 |
| EVDVSKKFAIQGFVK | 2 | 1693.94 | 0.00 | 849.48 | 49.31 |
| FAIQGFVK | 0 | 908.51 | 10032.28 | 26402.68 | 146.94 |
| KFAIQGFVK | 1 | 1036.61 | 0.00 | 717.99 | 87.25 |
| LTQQVETLTDSLK | 0 | 1474.79 | 0.00 | 597.90 | 88.02 |
| LTQQVETLTDSLKR | 1 | 1630.89 | 8566.50 | 50016.67 | 164.88 |
| NLLAGLLEGVR | 0 | 1153.68 | 0.00 | 1759.38 | 91.58 |
| SLLDVPDNLER | 0 | 1269.66 | 15993.75 | 46756.94 | 123.86 |
| TLAEMENLR | 0 | 1075.53 | 6325.10 | 4500.56 | 85.68 |
| TLAEMENLRAR | 1 | 1302.67 | 0.00 | 1464.86 | 48.74 |
| VIRPAEVGVVR | 1 | 1193.72 | 25326.71 | 44810.06 | 151.26 |
|  |  |  | 70819.3 | 192703.1 |  |
